# L-Carnitine Stimulates In Vivo Carbohydrate Metabolism in the Type 1 Diabetic Heart as Demonstrated by Hyperpolarized MRI

**DOI:** 10.3390/metabo11030191

**Published:** 2021-03-23

**Authors:** Dragana Savic, Vicky Ball, M. Kate Curtis, Maria da Luz Sousa Fialho, Kerstin N. Timm, David Hauton, James West, Julian Griffin, Lisa C. Heather, Damian J. Tyler

**Affiliations:** 1 Cardiac Metabolism Research Group, Department of Physiology Anatomy and Genetics, University of Oxford, Oxford OX1 3PT, UK; vicky.ball@dpag.ox.ac.uk (V.B.); kate.curtis@dpag.ox.ac.uk (M.K.C.); mariadaluz.fialho@gmail.com (M.d.L.S.F.); kerstin.timm@pharm.ox.ac.uk (K.N.T.); davehauton@gmail.com (D.H.); lisa.heather@dpag.ox.ac.uk (L.C.H.); damian.tyler@dpag.ox.ac.uk (D.J.T.); 2 Oxford Centre for Clinical Magnetic Resonance Research (OCMR), Division of Cardiovascular Medicine, Radcliffe Department of Medicine, University of Oxford, Oxford OX1 3PT, UK; 3 Department of Pharmacology, University of Oxford, Oxford OX1 3PT, UK; 4 Metabolomics Research Group, Department of Chemistry, University of Oxford, Oxford OX1 3PT, UK; 5 Department of Medicine, University of Cambridge, Cambridge CB2 1TN, UK; jaw68@medschl.cam.ac.uk; 6 Department of Metabolism, Digestion and Reproduction, Faculty of Medicine, Imperial College London, London SW7 2AZ, UK; julian.griffin@imperial.ac.uk

**Keywords:** magnetic resonance, L-carnitine, metabolic imaging, in vivo metabolism, hyperpolarized ^13^C, Langendorff perfusion, metabolomics, type-1 diabetes, cardiac imaging, streptozotocin

## Abstract

The diabetic heart is energetically and metabolically abnormal, with increased fatty acid oxidation and decreased glucose oxidation. One factor contributing to the metabolic dysfunction in diabetes may be abnormal handling of acetyl and acyl groups by the mitochondria. L-carnitine is responsible for their transfer across the mitochondrial membrane, therefore, supplementation with L-carnitine may provide a route to improve the metabolic state of the diabetic heart. The primary aim of this study was to use hyperpolarized magnetic resonance imaging (MRI) to investigate the effects of L-carnitine supplementation on the in vivo metabolism of [1-^13^C]pyruvate in diabetes. Male Wistar rats were injected with either vehicle or streptozotocin (55 mg/kg) to induce type-1 diabetes. Three weeks of daily i.p. treatment with either saline or L-carnitine (3 g/kg/day) was subsequently undertaken. In vivo cardiac function and metabolism were assessed with CINE and hyperpolarized MRI, respectively. L-carnitine supplementation prevented the progression of hyperglycemia, which was observed in untreated streptozotocin injected animals and led to reductions in plasma triglyceride and ß-hydroxybutyrate concentrations. Hyperpolarized MRI revealed that L-carnitine treatment elevated pyruvate dehydrogenase flux by 3-fold in the diabetic animals, potentially through increased buffering of excess acetyl-CoA units in the mitochondria. Improved functional recovery following ischemia was also observed in the L-carnitine treated diabetic animals.

## 1. Introduction

Patients with diabetes are at increased risk of cardiovascular disease (hazard ratio of 3.6–7.7 in patients with type-1 diabetes [1] and 1–5 in patients with type-2 diabetes [2]. The high energetic demands of the heart mean that dysregulated substrate utilization and mitochondrial impairment are likely to be contributing factors to the impaired cardiac function seen in the diabetic population [3,4]. The healthy heart generates 60–70% of its required adenosine triphosphate (ATP) production from fatty acids (FAs), with the remainder coming from glucose, amino-acids, ketone bodies and lactate [5]. However, in diabetes this balance is shifted to even higher rates of fatty acid oxidation [6,7]. Lipotoxicity has been suggested as a mechanistic link between the elevated supply and oxidation of fatty acids in the diabetic heart and the subsequent cardiac dysfunction, with excess lipids also being implicated in the decreased post-ischemic recovery observed in the diabetic heart. It has been proposed that an imbalance between the supply and oxidation of fatty acids may lead to a build-up of potentially toxic fatty acid intermediates (e.g., ceramide, diacylglycerol, etc.) in the cytosol, which leads to cardiovascular disease [8].

One potential reason for this imbalance may be an insufficient capacity for the handling of acetyl and acyl groups into and out of the mitochondria in the diabetic heart. Long-chain acyl groups are transported into mitochondria via the carnitine shuttle. The carnitine shuttle requires L-carnitine to accept the FA moiety from long-chain acyl-CoAs found in the cytoplasm, making them a suitable substrate for mitochondrial uptake via the carnitine-acylcarnitine translocase (CACT) within the inner mitochondrial membrane. It has previously been shown that plasma levels of L-carnitine are reduced in both diabetes [9,10,11] and cardiovascular disease [12,13]. Specifically, patients with type-1 diabetes (T1D) have been shown to have lower free and total plasma L-carnitine levels compared to the healthy population [9,10,13], a problem that worsens with the duration of the disease. In addition, several groups have found that myocardial acetyl-CoA and acyl-CoA levels are elevated in diabetes [14,15].

Supplementation with L-carnitine may therefore offer some therapeutic benefit to the diabetic population. Indeed, multiple beneficial effects have already been observed with L-carnitine supplementation in human patients with diabetes, such as reduced blood pressure [16], a decline in inflammation [17] and improved cardiac function [18,19].

However, no studies to-date have investigated the effects of L-carnitine treatment on in vivo metabolism. Therefore, in this study we aimed to further investigate the therapeutic potential of L-carnitine supplementation on the diabetic heart to explore the mechanism for its proposed beneficial effects. In vivo cardiac metabolism can be investigated using the novel technique of hyperpolarized MRI. Hyperpolarized MRI allows for >10,000-fold increases in the sensitivity of MRI to detect metabolic tracers labelled with the non-radioactive isotope, carbon-13 [20]. In this way, hyperpolarized MRI allows the instantaneous assessment of substrate uptake and cardiac metabolism in vivo in real time. By using hyperpolarized [1-^13^C]pyruvate, we aimed to investigate the effect of carnitine supplementation on in vivo pyruvate metabolism in the diabetic heart and to compare that to alterations in cardiac structure and function using CINE MRI. In addition, we also utilized a Langendorff perfusion method to explore the effect of L-carnitine supplementation on post-ischemic recovery in the diabetic heart.

## 2. Results

### 2.1. Animal Characterization

**Figure 1:**
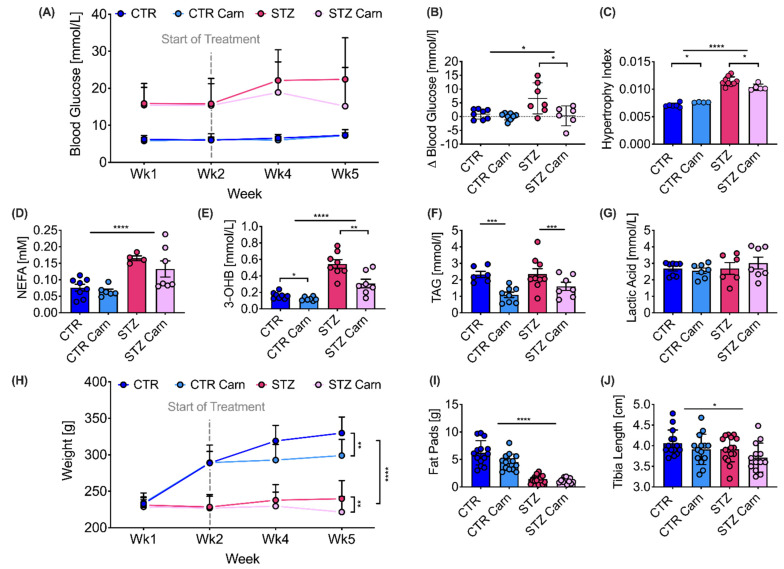
Carnitine induced changes in diabetes. Animal characteristics five weeks post streptozotocin-induced diabetes (STZ) or in citrate buffer injected controls (CTR), 50% of all animals were treated with L-carnitine (Carn) initiated at week 2. (**A**) Fasted blood glucose concentration at 1, 2, 4 and 5 weeks, **** between CTR and STZ groups at 1, 2, 4 and 5 weeks. (**B**) Delta blood glucose concentrations between week two and week five. (**C**) Kidney hypertrophy index assessed as kidney weight/body weight. (**D**) Non-esterified fatty acids (NEFA). (**E**) ß-hydroxybutyrate (3-OHB). (**F**) Triglycerides (TAG). (**G**) Lactic acid concentration. (**H**) Body weight measured at 1, 2, 4 and 5 weeks (Wk) post induction of diabetes. (**I**) Epididymal fat pads. (**J**) Tibia length. Data are presented as mean ± SD. Two-way ANOVA, if an interaction was found to be significant, the effect of L-carnitine on control and diabetes was evaluated using Sidak’s multiple comparison test. Significant differences are represented by ‘*’, with * *p* < 0.05, ** *p* < 0.01, *** *p* < 0.001, **** *p* < 0.0001.

Streptozotocin (STZ) injection in male Wistar rats led to fasting hyperglycemia (>15 mmol/L), observed at one-week post STZ injection that gradually increased throughout the course of the experiment (Figure 1A). At five weeks post STZ injection, diabetic animals had markedly elevated blood glucose levels, which was associated with an elevated hypertrophy index of the kidneys (Figure 1B,C) and an elevation of plasma non-esterified fatty acid (NEFA) levels by 111% and ß-hydroxybutyrate levels by 201%, while triglycerides and lactic acid levels remained unchanged (Figure 1D–G).

Diabetic animals failed to gain weight over the course of the study, leading to a significant difference in body weight between controls and diabetics at all time-points after the initial weight matching (Figure 1H). Lack of weight gain in the diabetic animals was attributed primarily to a reduction in fat mass as indicated by a 4.2-fold reduction in epididymal fat pad weight (Figure 1I). There was also a small, but significant, reduction in lean mass as measured by a decreased tibia length at the terminal time-point in the diabetic animals (Figure 1J).

Animals treated with L-carnitine had significantly lower body weights in both control and diabetic groups (Figure 1H). The progressive hyperglycemia seen in the saline treated STZ animals was not observed in the L-carnitine treated STZ animals, where the blood glucose levels did not continue to increase following the start of treatment (Figure 1A,B). L-carnitine supplementation significantly reduced the hypertrophy index in the STZ animals whilst it led to a small but significant increase in the control animals (Figure 1C). In addition, L-carnitine treatment reduced ß-hydroxybutyrate levels by 25% in the control animals and by 43% in the STZ animals and reduced triglyceride levels by 53% in the control animals and by 32% in the STZ animals (Figure 1E,F).

### 2.2. Cardiac Function

**Figure 2:**
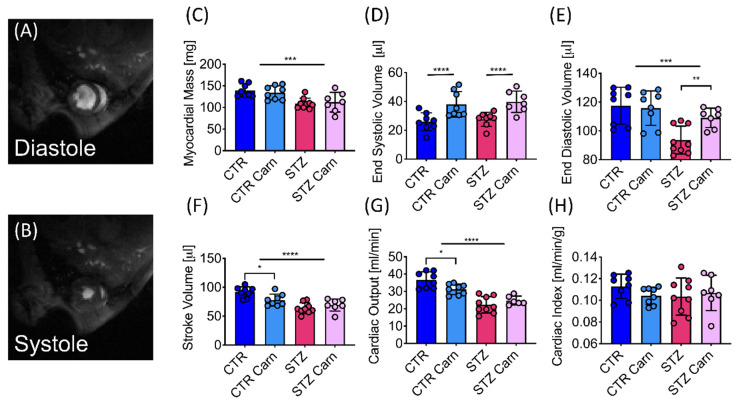
The diabetic heart displays reduced cardiac function on CINE magnetic resonance imaging (MRI). (**A**) Example CINE-MRI image of the rat heart in diastole. (**B**) Example CINE-MRI image of the rat heart at systole. (**C**) Average myocardial wall mass. (**D**) End-systolic volume (ESV). (**E**) End-diastolic volume (EDV). (**F**) Stroke volume (SV). (**G**) Cardiac output (CO). (**H**) Cardiac index. Data are presented as mean ± SD. Two-way ANOVA, if an interaction was found to be significant, the effect of L-carnitine on control and diabetes was evaluated using Sidak’s multiple comparison test. Significant differences are represented by ‘*’, with * *p* < 0.05, ** *p* < 0.01, *** *p* < 0.001, **** *p* < 0.0001.

Myocardial mass was reduced by 19% in the diabetic animals compared to the control animals (Figure 2C), a reduction that was slightly less than the reduction in body weight in the diabetic animals, leading to a small but significant increase in the heart weight/body weight ratio (Table 1). STZ-induced diabetes led to a 13% reduction in end-diastolic volume (EDV), which resulted in a 22% reduction in stroke volume (SV), and when combined with a significant reduction in heart rate (Table 1), a 31% reduction in cardiac output (Figure 2E–G). However, after normalization for the observed differences in body weight, no significant differences in cardiac index were seen across the groups (Figure 2H).

Animals treated with L-carnitine demonstrated elevated end-systolic volumes by 47% and 44% in the control and STZ injected animals, respectively (Figure 2D), leading to a significant reduction in ejection fraction (Table 1), potentially indicating a degree of systolic dysfunction. This change was highlighted by a 15% reduction in stroke volume and cardiac output in the L-carnitine treated control animals (Figure 2F), however, no further reduction in stroke volume or cardiac output was observed in the STZ animals treated with L-carnitine due to a significant increase in end-diastolic volume caused by L-carnitine supplementation (Figure 2E).

### 2.3. Cardiac Metabolism

**Figure 3:**
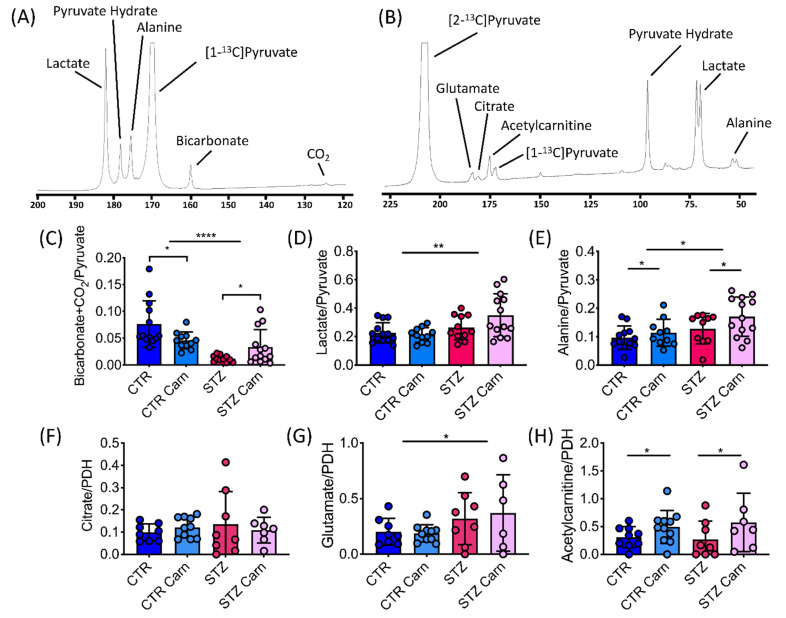
Carnitine can modulate in vivo cardiac metabolism. In vivo effects on cardiac metabolism by infusion of hyperpolarized [1-^13^C]pyruvate and [2-^13^C]pyruvate. (**A**) Example [1-^13^C]pyruvate spectrum acquired from the in vivo rat heart. (**B**) Example [2-^13^C]pyruvate spectrum acquired from the in vivo rat heart. (**C**) Bicarbonate + CO_2_/pyruvate ratio, as a measure of pyruvate dehydrogenase (PDH) flux. (**D**) Lactate/pyruvate ratio. (**E**) Alanine/pyruvate ratio. (**F**) Citrate/pyruvate ratio normalized to PDH flux. (**G**) Glutamate/pyruvate ratio normalized to PDH-flux. (**H**) Acetylcarnitine/pyruvate ratio normalized to PDH-flux. Data are presented as mean ± SD. Two-way ANOVA, if an interaction was found to be significant, the effect of L-carnitine on control and diabetes was evaluated using Sidak’s multiple comparison test. Significant differences are represented by ‘*’, with * *p* < 0.05, ** *p* < 0.01, **** *p* < 0.0001.

As has previously been observed [21], pyruvate dehydrogenase (PDH) flux, as assessed by ^13^C-bicarbonate and ^13^CO_2_ production from the injected hyperpolarized [1-^13^C]pyruvate, was significantly reduced in the STZ-induced diabetic heart (Figure 3C). L-carnitine treatment led to a differential response in the control and diabetic animals, causing a significant increase in PDH flux, by approximately 3-fold in the diabetic animals and a significant decrease in PDH flux in the control animals by 1.7-fold. The diabetic animals showed elevated production of lactate and alanine compared to the controls (Figure 3D,E), and L-carnitine treatment led to a small but significant elevation in alanine production in both the control (18%) and the diabetic (33%) heart (Figure 3E).

Following injection of hyperpolarized of [2-^13^C]pyruvate, and after normalization for differences in PDH flux, no changes were observed in the incorporation of the ^13^C label into citrate across the groups (Figure 3F). However, a small but significant increase in ^13^C label incorporation into glutamate was observed in the STZ injected animals (Figure 3G). L-carnitine treatment led to a 1.6-fold and 2.1-fold increase in the incorporation of the ^13^C label into acetylcarnitine in the control and diabetic animals respectively (Figure 3H).

### 2.4. Carnitine/Acyl Carnitine Levels

**Figure 4:**
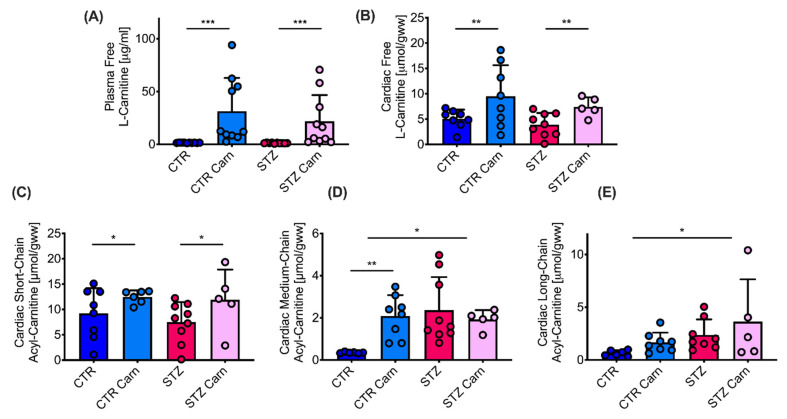
Carnitine modulates cardiac acyl-carnitine species. (**A**) Free L-carnitine in plasma. (**B**) Free L-carnitine in cardiac tissue. (**C**) All short-chain acyl-carnitines summed (C2-C10) in heart tissue. (**D**) All medium-chain acyl-carnitines summed (C12–C15) in heart tissue. (**E**) All long-chain acyl-carnitines summed (>C15) in heart tissue. Data are presented as mean ± SD. Two-way ANOVA, if an interaction was found to be significant, the effect of L-carnitine on control and diabetes was evaluated using Sidak’s multiple comparison test. Significant differences are represented by ‘*’, with * *p* < 0.05, ** *p* < 0.01, *** *p* < 0.001.

STZ animals showed no significant differences in either plasma or cardiac free L-carnitine levels when compared to control animals (Figure 4A,B). Equally there were no differences in short-chain acyl-carnitine levels between STZ and CTR animals when all species were summed together (Figure 4C); however, when the individual carnitine species were considered separately (Table 2), significant increases in C5, C6, C8, C8_1 and C10 acyl-carnitines were observed in the hearts of STZ injected animals. When considering the medium- and long-chain acyl-carnitines (Figure 4D,E), significant increases were also observed in the STZ animals compared to the control animals (Figure 4D,E).

L-carnitine treatment elevated both plasma and cardiac free L-carnitine levels, as well as short-chain acyl-carnitine levels in the cardiac tissue of control and STZ animals (Figure 4A–C). Analysis of the individual short-chain carnitine species, showed this elevation to be driven by significant increases in the C3, C4, C5, C6 and C8 species (Table 2). L-carnitine treatment led to a significant increase in the medium-chain acyl-carnitines in the control animals but had no effect on the long-chain acyl-carnitines in either the STZ or control animals (Figure 4D,E).

### 2.5. Post-Ischemic Recovery

**Figure 5:**
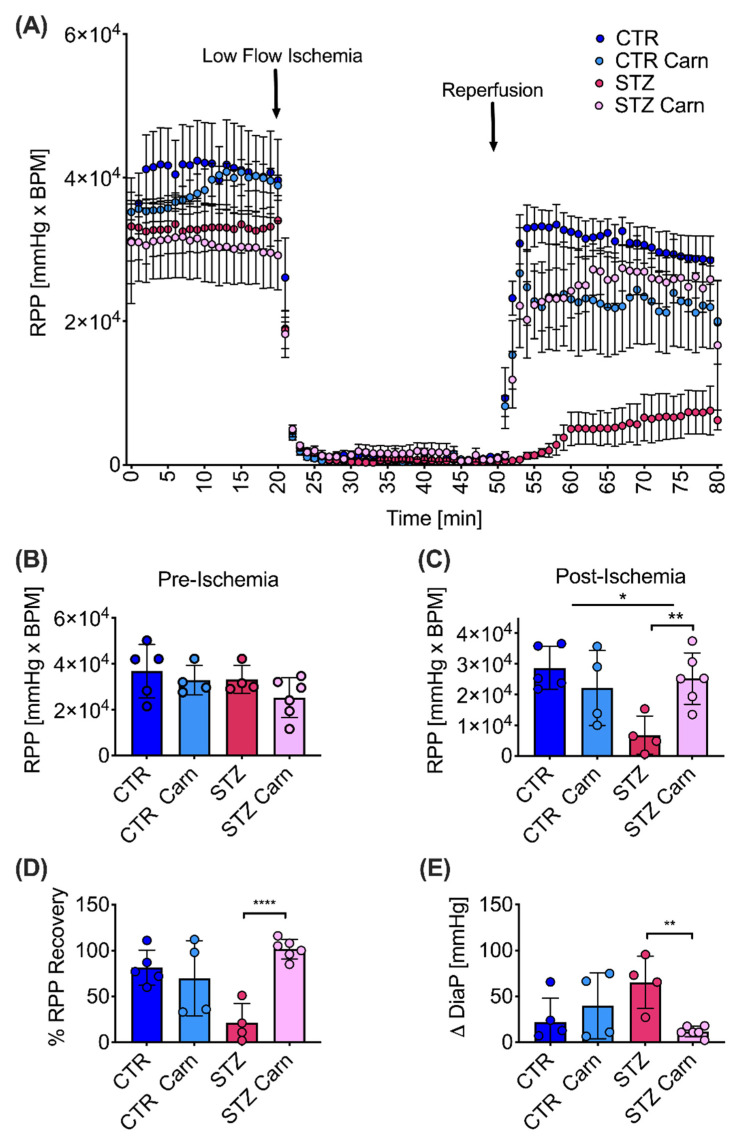
Carnitine rescues cardiac function in diabetes after low-flow ischemia in Langendorff perfused hearts. (**A**) Rate pressure product (RRP) over time: pre-ischemia (t = 1:20 min), low flow ischemia (t = 21:50 min), post ischemia (t = 51:80 min). (**B**) Rate pressure product (RRP) pre-ischemia. (**C**) Rate pressure product (RRP) post-ischemia. (**D**) Percentage recovery between pre-ischemia (t = 10:20 min) and post-ischemia (t = 70:80 min). (**E**) Difference between post-ischemia and pre-ischemic diastolic pressure (∆DiaP). Diastolic pressure was set during pre-ischemia to 4–8 mmHg. Data are presented as mean ± SD. Two-way ANOVA, if an interaction was found to be significant, the effect of L-carnitine on control and diabetes was evaluated using Sidak’s multiple comparison test. Significant differences are represented by ‘*’, with * *p* < 0.05, ** *p* < 0.01, **** *p* < 0.0001.

As a marker of cardiac function, rate pressure product (RPP) was similar across all groups pre-ischemia. However, a functional impairment was observed post-ischemia with RPP 76% lower in the diabetic hearts after reperfusion compared with controls (Figure 5A–C). The observed impairment in post-ischemic function in the diabetic heart was due to a reduced systolic pressure (26% reduction) and developed pressure (67% reduction) when compared with the control animals (Table 3). However, L-carnitine treatment led to a significant elevation in post-ischemic RPP by 3.6-fold in the L-carnitine treated diabetic animals (Figure 5C). The improved functional recovery post ischemia (Figure 5D) was driven by a significantly reduced difference (delta) in the diastolic pressure between pre- and post-ischemia in the L-carnitine treated diabetic animals (Figure 5E).

## 3. Discussion

The primary aim of this work was to explore the metabolic and functional effects of L-carnitine supplementation on the diabetic heart. In line with previous studies [19,22], daily L-carnitine injections led to improvements in blood glucose levels, preventing the progressive hyperglycemia seen in the untreated diabetic animals, and led to improved functional recovery post-ischemia, with increased RPP and reduced diastolic pressure. These beneficial effects occurred in parallel with increased in vivo flux through pyruvate dehydrogenase (PDH), indicating increased glucose utilization in the L-carnitine treated diabetic heart. However, L-carnitine supplementation also led to a reduction in ejection fraction in both control and diabetic animals, a finding that requires further investigation.

Daily L-carnitine treatment led to increased levels of free L-carnitine in the hearts of both the control and STZ injected animals, resulting in the increased incorporation of hyperpolarized [2-^13^C]pyruvate into [1-^13^C]acetylcarnitine. Increased availability of free L-carnitine by the treatment regimen was hypothesized to increase the buffering of excess fatty acid-derived acetyl-CoA and acyl-CoA units, which we expected would allow increased flux through PDH, known to be inhibited in diabetes [23,24]. This finding is supported by the metabolomic data, which revealed increased levels of short chain acyl-carnitine species in the hearts of the carnitine treated animals, with significant increases observed in the C3, C4, C5, C6 and C8 acyl-carnitines.

In the diabetic animals, L-carnitine supplementation led to increased flux of [1-^13^C]pyruvate through the pyruvate dehydrogenase complex, indicating increased glucose oxidation in the L-carnitine treated diabetic heart. This finding is supported by previous work, which has demonstrated increased flux through PDH in isolated rat cardiomyocytes [25] and increased glucose oxidation in perfused rat hearts acutely treated with L-carnitine [26,27]. It is also in agreement with previous observations in ex vivo isolated human skeletal muscle mitochondria [28].

In contrast to the effect seen in the diabetic heart, L-carnitine supplementation led to a significant reduction in flux through PDH in the healthy heart. The differential effect observed here may find its basis in the different levels of fatty acid oxidation that occur in the healthy and diabetic heart. In the setting of diabetes, the elevated levels of fatty acid oxidation are known to lead to inhibition of PDH through upregulation of PDH kinase expression and elevated levels of fatty acid-derived acetyl-CoA and NADH [23,24]. Providing supplemental L-carnitine may offer a buffer of these excess acetyl-CoA units, and therefore, a release of the fatty acid-derived inhibition of PDH [28]. In the healthy heart, the provision of additional L-carnitine may have the opposite effect, i.e., increasing the uptake of fatty acids into the mitochondria through the carnitine shuttle from normal baseline levels, increasing the rate of fatty acid oxidation and leading, as seen in the untreated diabetic heart, to inhibition of PDH due to the increased levels of fatty acid-derived acetyl-CoA and NADH.

As we have previously proposed, increasing flux through PDH can be considered as a potential therapeutic target in diabetic cardiomyopathy, leading to improved cardiac function [29]. In this study, functional improvement was seen in terms of increased RPP recovery post-ischemia in the Langendorff perfused heart. The improved RPP was mediated by an increase in developed pressure in the carnitine treated diabetic hearts, driven by reductions in the post-ischemic diastolic pressure. In agreement with this finding, previous work from our laboratory has demonstrated improvements in in vivo diastolic function following treatment with the PDH activator, dichloroacetate (DCA) [30].

However, assessment of in vivo cardiac function revealed a significant increase in end systolic function in the control and diabetic animals treated with L-carnitine, leading to a significant reduction in ejection fraction, indicating a degree of systolic dysfunction. In the control animals treated with L-carnitine, this also led to a significant reduction in stroke volume and cardiac output. These functional changes were seen in line with significant reductions in body weight in the L-carnitine treated animals and when the functional data was normalized for these body weight differences, no significant differences in cardiac index were observed. Many previous studies have reported beneficial effects of L-carnitine treatment on cardiac function; however, such reports have commonly explored the effects on cardiac function in ex vivo perfused hearts and only in diabetic animals treated with L-carnitine [19]. Further work is clearly required to investigate the effect of L-carnitine supplementation on cardiac function in the healthy heart.

L-carnitine supplementation has previously been proposed as a potential weight loss agent, with a meta-analysis in human subjects revealing that those who received L-carnitine lost significantly more weight [31]. The mechanism proposed for this weight loss is increasing energy expenditure caused by the effect of L-carnitine on glucose and lipid metabolism [32]. In addition, Rodrigues et al. showed that two weeks of L-carnitine treatment led to lower intake of food compared to normal rats [19], suggesting alleviation of perceived hunger [33]. Even though food was not weighed in this study, it seems likely that the decreased weight gain may be due to a combination of lower food consumption and the direct effects of L-carnitine on whole-body metabolism.

### Study Limitations

In addition to the observed blood glucose improvements, increased PDH flux, reduced kidney hypertrophy index and improved cardiac function post-ischemia, daily L-carnitine injections led to reductions in triglyceride and ß-hydroxybutyrate levels in the diabetic animals. Such findings are potentially indicative of changes in systemic lipid handling (for example, increased fatty acid oxidation, increased ketolysis or decreased de novo lipogenesis). Unfortunately, it was not possible to directly assess fatty acid oxidation in vivo, as hyperpolarized MRS is currently unable to probe the oxidation of long-chain fatty acids. However, recent developmental work has shown the ability of hyperpolarized MRS to probe the oxidation of short-chain fatty acids and ketone bodies [34,35,36], which may provide further mechanistic insight into the effects of L-carnitine supplementation.

Previous studies of STZ induced diabetes have revealed reductions in free L-carnitine in the diabetic heart [19]. In our study, the free L-carnitine levels in the saline-treated STZ injected diabetic animals were 3.90 ± 2.30 μmol/gww, a 23% reduction compared to the saline injected control animals at 5.05 ± 2.30 μmol/gww. However, this reduction failed to reach statistical significance (*p* = 0.24). It is possible that a longer duration of diabetes (in our study the animals were diabetic for five weeks versus the six week period investigated by Rodrigues et al. [19]) or larger group sizes may help to clarify this point.

## 4. Materials and Methods

### 4.1. Animal Protocol

Animal studies were conducted in accordance with the UK Animals (Scientific Procedures) Act (1986), PPL Number 30/3322, and local ethical guidelines (Medical Research Council Responsibility on the Use of Animals for Medical Research, July 1993). Seventy-two male Wistar rats (~200 g) were randomly divided into four groups. All animals were fasted overnight and then either made diabetic with one i.p. injection of streptozotocin (STZ, 55 mg/kg in citrate buffer) or injected with vehicle citrate buffer.

Two weeks after STZ/citrate buffer injection, all animals were initiated on daily morning intraperitoneal (i.p.) injections of either saline or L-carnitine (3 g/kg/day), for three weeks. After two weeks of treatment (four weeks after STZ/vehicle), all animals were anesthetized with 2.5% isoflurane in 1 liter/min O_2_ and subjected to MR imaging and hyperpolarized MR spectroscopy (MRS).

After three weeks of treatment, all animals were euthanized in the fed state with 5% isoflurane vol:vol in 2 liters/min O_2_, followed by removal of the heart and kidneys. The hypertrophy index (HI) was calculated as the sum of the left and right kidney weights normalized to body weight. Other investigators have reported HI in the literature as a progressive marker of diabetic renal disease [37,38,39]. The right tibia length was measured and epididymal fat pads were obtained from the posterior subcutaneous depots as described by Chusyd et al. [40]. In a subset of animals, the hearts were excised for Langendorff perfusion, whilst in the others, the hearts were immediately freeze-clamped in liquid nitrogen for biochemical analysis. Blood samples, taken from the chest cavity, were centrifuged, and plasma stored at -80 °C for later biochemical analysis.

### 4.2. CINE Magnetic Resonance Imaging (MRI)

Rodents were imaged on a 7T horizontal bore MRI instrument (Varian Inc, Santa Clara, CA, USA), using a four-channel ^1^H phased-array four-channel surface receive coil (RAPID Biomedical GmbH, Rimpar, Germany) and a 72 mm ^1^H/^13^C volume transmit coil. Eight to ten short-axis slices (slice thickness, 1.6 mm; matrix size, 128 × 128; TE/TR, 4.6/1.45 ms; flip angle, 18°; number of averages, 4) were acquired with a CINE-FLASH sequence [41]. Left ventricular volumes were derived using the free-hand draw function in ImageJ (NIH, Bethesda, MD, USA). For each heart, left ventricular mass, ejection fraction, stroke volume and cardiac output were calculated. Average myocardial wall mass of the left ventricle was obtained from the average of the end-diastolic and end-systolic masses. Stroke volume was obtained from the difference between the end-diastolic lumen and the end-systolic lumen.

### 4.3. Hyperpolarized Magnetic Resonance Spectroscopy (MRS)

Experiments were performed between 7 am and 1 pm, when rodents were in the fed state. Approximately 40 mg of either [1-^13^C]pyruvic acid or [2-^13^C]pyruvic acid (Sigma-Aldrich, St. Louis, MO, USA) doped with 15mM trityl radical (OXO63, GE Healthcare) and 3μL Dotarem (1:50 dilution, Guerbet), was hyperpolarized in a prototype polarizer, with 30–40 min of microwave irradiation [20]. The sample was subsequently dissolved in a pressurized and heated alkaline solution, containing 2.4 g/L sodium hydroxide and 100 mg/L EDTA dipotassium salt (Sigma-Aldrich, St. Louis, MO, USA), to yield an 80 mM solution of either hyperpolarized sodium [1-^13^C]pyruvate or [2-^13^C]pyruvate with a polarization of approximately 30% or 20% respectively, at physiological temperature and pH. From the resulting solution, 1 mL was injected over 10 s via a tail vein catheter into a rat located in the 7T MRI system described above. Using the 72 mm ^1^H/^13^C volume transmit coil and a two-channel ^13^C surface receive coil (RAPID Biomedical GmbH, Rimpar, Germany), ECG-gated, ^13^C MR pulse-acquire cardiac spectra were acquired over 60 s following injection of hyperpolarized pyruvate (repetition time, 1 s; excitation flip angle, 15°; sweep width, 13,021 Hz; acquired points, 2048) [42]. Each animal was injected with [1-^13^C]pyruvate and [2-^13^C]pyruvate in a random order and with at least one hour between injections. The ^13^C label from pyruvate and its metabolic products were summed over 30 s from the first appearance of pyruvate, and fitted with the AMARES algorithm within jMRUI [43]. Each of the metabolites was quantified as the ratio of the metabolite to either [1-^13^C]pyruvate or [2-^13^C]pyruvate. All metabolites obtained from [2-^13^C]pyruvate were subsequently normalized to pyruvate dehydrogenase (PDH) flux, measured as the ^13^C-bicarbonate + ^13^CO_2_/[1-^13^C]pyruvate ratio recorded in the same animal, in order to assess any changes within the Krebs cycle independent of any changes in ^13^C label flux through PDH.

### 4.4. Langendorff Perfusion

A Langendorff ischemia-reperfusion protocol was undertaken in a subset of animals, five weeks post induction of diabetes. Following excision, the hearts were immediately cannulated via the aorta and perfused with warm Krebs–Henseleit (KH) buffer (37 °C) containing 11 mM glucose and 0.4 mM of palmitate, at a constant pressure of 100 mmHg as described by Heather et al. [44]. A water-filled PVC balloon, which was connected via a polythene tube to a calibrated pressure transducer, a bridge amplifier, and a PowerLab data acquisition system (AD Instruments, Oxfordshire, UK), was inserted into the left ventricle to measure cardiac function. The balloon was inflated to an end-diastolic pressure of 4–8 mmHg. Developed pressure was calculated as the difference between systolic and diastolic pressure, and rate pressure product (RPP) was obtained by multiplying developed pressure by heart rate. Hearts were subjected to 20 min of normal flow (t = 1:20 min), followed by 30 min of low-flow ischemia (0.4 mL/min, t = 21:50 min) and reperfused again with normal flow for a further 30 min (t = 51:80 min). The hearts were freeze-clamped with liquid nitrogen-cooled Wallenberger tongs whilst still beating on the perfusion apparatus at t = 80 min.

### 4.5. Blood Metabolites

Fasted blood samples and glucose levels were obtained at two, four and five weeks post STZ injection. Insulin (Mercodia, Uppsala, Sweden) and NEFA (Randox Laboratories, Crumlin, County Antrim, UK) were measured in the fasted plasma using assay kits. Terminal fed plasma metabolites samples were analyzed for ß-hydroxybutyrate (3-OHB), triglycerides (TAG) and lactic acid using an ABX Pentra 400 (Horiba ABX-UK, Northampton, UK).

Terminal fed blood samples were also assessed for low-molecular-weight metabolites with liquid chromatography-mass spectrometry (LC-MS) within the Department of Chemistry, University of Oxford. Plasma samples were filtered through molecular weight cut-off filters (10 kDa) to remove proteins [45]. The infranatant was recovered and evaporated to dryness under reduced pressure. Sample residue was then resuspended in acetonitrile:water (95%:5%). For LC-MS, acylcarnitines were separated and resolved using hydrophobic-interaction liquid chromatography-mass spectrometry (HILIC) as previously outlined [45,46]. Putative compounds were identified with reference to authenticated standards for selected acylcarnitines using retention time, accurate mass and fragmentation pattern to identify individual compounds [45]. Concentrations were calculated with reference to specific standard curves.

### 4.6. Cardiac Tissue Metabolomics

On the freeze-clamped non-perfused hearts, low-molecular-weight metabolites were assessed using Liquid Chromatography with tandem Mass Spectrometry (LC-MS/MS) as previously described [47]. Tissue extraction of freeze-clamped hearts was done at 30 mg/500 μL MeOH:Chloroform (2:1 *v*/*v*), using metal-bead containing tubes and a Precellys tissue homogenizer (Bertin Instruments, Montigny-le-Bretonneux, France), at 6000 rpm for 2 × 30s with a 2 min rest on ice in between each cycle. Pellets were extracted twice and lipid and aqueous phases evaporated separately, dried extracts were then resuspended just prior to LC-MS/MS as described by Wang et al. [47]. Metabolomics of aqueous metabolites in heart tissue extracts were subsequently summed into short, medium and long-chain acyl-carnitines. Short chain acyl-carnitines contained a carbon chain length of 10 carbons or less, medium chain acyl-carnitines were between 12 and 15 carbons in length, and long chain acyl-carnitines were longer than 15 carbon molecules.

### 4.7. Statistics

All data are presented as mean ± standard deviation (SD) of the indicated number of rodents (n). Two-way ANOVA was used for assessment of significance examining the effects of STZ injection and the effect of L-carnitine treatment. When an interaction term was significant in the two-way ANOVA, post-hoc multiple comparison testing using Sidak’s correction was used to investigate the effect of L-carnitine supplementation on both the control and diabetic groups, respectively. Outlier analysis was undertaken using Grubb’s test in Graphpad Prism (GraphPad Software, San Diego, CA, USA). Differences between groups were considered statistically significant if *p* < 0.05.

## 5. Conclusions

In summary, we have demonstrated that L-carnitine treatment in the diabetic heart leads to improved glucose handling, reduced levels of kidney hypertrophy and improved functional recovery following ischemia-reperfusion, as assessed by increased RPP and reduced diastolic pressure. Mechanistically, these outcomes are potentially linked with an L-carnitine driven increase in PDH flux due to buffering of short chain acyl carbons within the myocardium of the diabetic heart. However, significant reductions in ejection fraction were observed following L-carnitine treatment, which requires further investigation.

## Figures and Tables

**Table 1:** Cardiac function obtained from CINE-MRI. Functional parameters are normalized to body weight (BW), where stated. Data presented as mean ± SD. Two-way ANOVA. *p*-value displayed with the effect of streptozotocin (STZ), the effect of L-carnitine (Carn) and the interaction between the two effects.

	Control (CTR)		Streptozotocin (STZ)		STZ	Carn	Interaction
	Saline	L-Carnitine	Saline	L-Carnitine	*p*-Value	*p*-Value	*p*-Value
Myocardial mass/BW	0.43 ± 0.06	0.45 ± 0.06	0.51 ± 0.07	0.52 ± 0.07	**0.002**	0.57	0.94
Heart Rate (HR)	408 ± 15	405 ± 41	353 ± 33	335 ± 40	**<0.0001**	0.38	0.51
End-Systolic Volume/BW	0.08 ± 0.02	0.13 ± 0.03	0.13 ± 0.03	0.19 ± 0.06	**0.0003**	**0.0002**	0.49
End-Diastolic Volume/BW	0.36 ± 0.04	0.39 ± 0.04	0.42 ± 0.04	0.52 ± 0.05	**<0.0001**	**0.0003**	**0.018**
Stroke Volume/BW (Stroke Index)	0.28 ± 0.02	0.26 ± 0.03	0.30 ± 0.04	0.32 ± 0.03	**0.0008**	0.87	**0.024**
Ejection Fraction (EF)	78 ± 4	67 ± 6	68 ± 8	63 ± 8	**0.0057**	**0.0024**	0.20

**Table 2:** Short-chain cardiac acyl-carnitine levels for the different species assessed. Data are presented as mean ±SD. Two-way ANOVA. *p*-value displayed with the effect of streptozotocin (STZ), the effect of L-carnitine (Carn) and the interaction between the two effects.

	Control (CTR)		Streptozotocin (STZ)		STZ	Carn	Interaction
	Saline	L-Carnitine	Saline	L-Carnitine	*p*-Value	*p*-Value	*p*-Value
C2 (μmol/gww)	8.6 ± 4.9	11.3 ± 1.7	6.8 ± 3.8	10.1 ± 5.3	0.33	0.07	0.82
C3 (μmol/gww)	0.30 ± 0.11	0.73 ± 0.34	0.26 ± 0.14	1.09 ± 0.59	0.17	**<0.0001**	0.10
C4 (μmol/gww)	0.11 ± 0.03	0.39 ± 0.17	0.20 ± 0.09	0.36 ± 0.18	0.56	**<0.0001**	0.17
C5 (μmol/gww)	0.04 ± 0.02	0.12 ± 0.08	0.06 ± 0.04	0.24 ± 0.13	**0.02**	**<0.0001**	0.11
C5_1 (μmol/gww)	0.007 ± 0.003	0.010 ± 0.004	0.005 ± 0.003	0.007 ± 0.005	0.10	0.05	0.88
C6 (μmol/gww)	0.009 ± 0.004	0.055 ± 0.033	0.054 ± 0.034	0.089 ± 0.042	**0.002**	**0.002**	0.61
C8 (μmol/gww)	0.002 ± 0.001	0.010 ± 0.003	0.018 ± 0.012	0.024 ± 0.011	**0.0002**	**0.04**	0.85
C8_1 (μmol/gww)	7.0 ± 1.5 × 10^−5^	4.9 ± 2.5 × 10^−5^	8.2 ± 4.2 × 10^−5^	10.9 ± 3.2 × 10^−5^	**0.02**	0.82	0.11
C10 (μmol/gww)	0.002 ± 0.001	0.008 ± 0.002	0.015 ± 0.007	0.018 ± 0.011	**<0.001**	0.06	0.54
C10_1 (μmol/gww)	0.0002 ± 0.0001	0.0002 ± 0.0001	0.0003 ± 0.0001	0.0002± 0.0001	0.52	0.44	0.71

**Table 3:** Langendorff perfusions. Functional parameters obtained pre- and post-ischemia in control (CTR) and diabetic (STZ) animals using a Langendorff ischemia-reperfusion method. Data are presented as mean ± SD. Two-way ANOVA. *p*-value displayed with the effect of streptozotocin (STZ), the effect of L-carnitine (Carn) and the interaction between these effects. STZ = streptozotocin, Carn = carnitine, CTR = control, RPP = rate pressure product, HR = heart rate.

Pre-Ischemia							
	Control (CTR)		Streptozotocin (STZ)		STZ	Carn	Interaction
	Saline	L-Carnitine	Saline	L-Carnitine	*P*-Value	*p*-Value	*p*-Value
RPP (×10^4^ mmHg × bpm)	3.7 ± 1.2	3.3 ± 0.6	3.3 ± 0.6	2.5 ± 0.9	0.19	0.17	0.63
HR (bpm)	290 ± 42	227 ± 41	221 ± 20	195 ± 38	**0.010**	**0.021**	0.30
Developed Pressure (mmHg)	132 ± 38	137 ± 12	149 ± 15	125 ± 31	0.87	0.48	0.29
Systolic Pressure (mmHg)	133 ± 33	141 ± 10	148 ± 19	134 ± 25	0.74	0.77	0.32
**Post-Ischemia**							
	**Control (CTR)**		**Streptozotocin (STZ)**		**STZ**	**Carn**	**Interaction**
	**Saline**	**L-Carnitine**	**Saline**	**L-Carnitine**	***p*-Value**	***p*-Value**	***p*-Value**
RPP (×10^4^ mmHg × bpm)	2.9 ± 0.7	2.2 ± 1.2	0.7 ± 0.6	2.5 ± 0.8	**0.034**	0.16	**0.0072**
HR (bpm)	256 ± 50	201 ± 28	197 ± 103	253 ± 48	0.91	0.99	0.072
Developed Pressure (mmHg)	115 ± 34	106 ± 46	38 ± 43	106 ± 14	**0.028**	0.085	**0.029**
Systolic Pressure (mmHg)	137 ± 31	149 ± 18	102 ± 19	124 ± 12	**0.0078**	0.10	0.63
Diastolic Pressure (mmHg)	22 ± 24	43 ± 35	65 ± 30	14 ± 5	0.60	0.23	**0.009**

## Data Availability

The data presented in this study are available on request from the corresponding author.

## References

[1] Soedamah-Muthu S.S., Fuller J.H., Mulnier H.E., Raleigh V.S., Lawrenson R.A., Colhoun H.M. (2006). High Risk of Cardiovascular Disease in Patients With Type 1 Diabetes in the U.K.: A cohort study using the General Practice Research Database. Diabetes Care.

[2] Leon B.M. (2015). Diabetes and cardiovascular disease: Epidemiology, biological mechanisms, treatment recommendations and future research. World J. Diabetes.

[3] Isfort M., Stevens S.C.W., Schaffer S., Jong C.J., Wold L.E. (2013). Metabolic dysfunction in diabetic cardiomyopathy. Heart Fail. Rev..

[4] Scheuermann-Freestone M., Madsen P.L., Manners D., Blamire A.M., Buckingham R.E., Styles P., Radda G.K., Neubauer S., Clarke K. (2003). Abnormal Cardiac and Skeletal Muscle Energy Metabolism in Patients With Type 2 Diabetes. Circulation.

[5] Murashige D., Jang C., Neinast M., Edwards J.J., Cowan A., Hyman M.C., Rabinowitz J.D., Frankel D.S., Arany Z. (2020). Comprehensive quantification of fuel use by the failing and nonfailing human heart. Science.

[6] Doenst T., Nguyen T.D., Abel E.D. (2013). Cardiac metabolism in heart failure: Implications beyond ATP production. Circ. Res..

[7] Lopaschuk G.D., Ussher J.R., Folmes C.D.L., Jaswal J.S., Stanley W.C. (1960). Myocardial metabolism of fatty acids. J. Clin. Investig..

[8] Schulze P.C., Drosatos K., Goldberg I.J. (2016). Lipid Use and Misuse by the Heart. Circ. Res..

[9] la Marca G., Malvagia S., Toni S., Piccini B., Di Ciommo V., Bottazzo G.F. (2013). Children who develop type 1 diabetes early in life show low levels of carnitine and amino acids at birth: Does this finding shed light on the etiopathogenesis of the disease?. Nutr. Diabetes.

[10] Mamoulakis D., Galanakis E., Dionyssopoulou E., Evangeliou A., Sbyrakis S. (2004). Carnitine deficiency in children and adolescents with type 1 diabetes. J. Diabetes Complicat..

[11] Savic D., Ball V., Holzner L., Hauton D., Timm K.N., Curtis M.K., Heather L.C., Tyler D.J. (2021). Hyperpolarized magnetic resonance shows that the anti-ischemic drug meldonium leads to increased flux through pyruvate dehydrogenase in vivo resulting in improved post-ischemic function in the diabetic heart. NMR Biomed..

[12] DiNicolantonio J.J., Lavie C.J., Fares H., Menezes A.R., O’Keefe J.H. (2013). L-Carnitine in the Secondary Prevention of Cardiovascular Disease: Systematic Review and Meta-analysis. Mayo Clin. Proc..

[13] Wittels B., Bressler R. (1964). Biochemical Lesion of Diphtheria Toxin in the Heart. J. Clin. Investig..

[14] Mansor L.S., Fialho M.D.L.S., Yea G., Coumans W.A., West J.A., Kerr M., Carr C.A., Luiken J.J., Glatz J.F., Evans R.D. (2017). Inhibition of sarcolemmal FAT/CD36 by sulfo-N-succinimidyl oleate rapidly corrects metabolism and restores function in the diabetic heart following hypoxia/reoxygenation. Cardiovasc. Res..

[15] Kanter J.E., Kramer F., Barnhart S., Averill M.M., Vivekanandan-Giri A., Vickery T., Li L.O., Becker L., Yuan W., Chait A. (2012). Diabetes promotes an inflammatory macrophage phenotype and atherosclerosis through acyl-CoA synthetase 1. Proc. Natl. Acad. Sci. USA.

[16] Miguel-Carrasco J.L., Mate A., Monserrat M.T., Arias J.L., Aramburu O., Vázquez C.M. (2008). The Role of Inflammatory Markers in the Cardioprotective Effect of L-Carnitine in L-NAME-Induced Hypertension. Am. J. Hypertens..

[17] Duranay M., Akay H., Yilmaz F.M., Şeneş M., Tekeli N., Yücel D. (2006). Effects of L-carnitine infusions on inflammatory and nutritional markers in haemodialysis patients. Nephrol. Dial. Transplant..

[18] Iliceto S., Scrutinio D., Bruzzi P., D’Ambrosio G., Boni A., Di Biase M., Biasco G., Hugenholtz P., Rizzon P. (1995). Effects of l-carnitine administration on left ventricular remodeling after acute anterior myocardial infarction: The l-Carnitine Ecocardiografia Digitalizzata Infarto Miocardico (CEDIM) trial. J. Am. Coll. Cardiol..

[19] Rodrigues B., Xiang H., Mcneill J.H. (1988). Effect of L-Carnitine Treatment on Lipid Metabolism and Cardiac Performance in Chronically Diabetic Rats. Diabetes.

[20] Ardenkjaer-Larsen J.H., Fridlund B., Gram A., Hansson L., Lerche M.H., Servin R., Thaning M., Golman K. (2003). Increase in signal-to-noise ratio of >10,000 times in liquid-state NMR. Proc. Natl. Acad. Sci. USA.

[21] Schroeder M.A., Cochlin L.E., Heather L.C., Clarke K., Radda G.K., Tyler D.J. (2008). In vivo assessment of pyruvate dehydrogenase flux in the heart using hyperpolarized carbon-13 magnetic resonance. Proc. Natl. Acad. Sci. USA.

[22] Paulson D.J., Schmidt M.J., Traxler J.S., Ramacci M.T., Shug A.L. (1984). Improvement of myocardial function in diabetic rats after treatment with L-carnitine. Metabolism.

[23] Seymour A.-M.L., Chatham J.C. (1997). The Effects of Hypertrophy and Diabetes on Cardiac Pyruvate Dehydrogenase Activity. J. Mol. Cell. Cardiol..

[24] Rohm M., Savic D., Ball V., Curtis M.K., Bonham S., Fischer R., Legrave N., Macrae J.I., Tyler D.J., Ashcroft F.M. (2018). Cardiac Dysfunction and Metabolic Inflexibility in a Mouse Model of Diabetes Without Dyslipidemia. Diabetes.

[25] Abdel-Aleem S., Sayed-Ahmed M., Nada M.A., Hendrickson S.C., St Louis J., Lowe J.E. (1995). Stimulation of non-oxidative glucose utilization by L-carnitine in isolated myocytes. J. Mol. Cell Cardiol..

[26] Broderick T., Quinney H., Lopaschuk G. (1992). Carnitine stimulation of glucose oxidation in the fatty acid perfused isolated working rat heart. J. Biol. Chem..

[27] Broderick T.L., Quinney H.A., Lopaschuk G.D. (1995). L-carnitine increases glucose metabolism and mechanical function following ischaemia in diabetic rat heart. Cardiovasc. Res..

[28] Uziel G., Garavaglia B., Di Donato S. (1988). Carnitine stimulation of pyruvate dehydrogenase complex (PDHC) in isolated human skeletal muscle mitochondria. Muscle Nerve.

[29] Lewis A.J., Neubauer S., Tyler D.J., Rider O.J. (2015). Pyruvate dehydrogenase as a therapeutic target for obesity cardiomyopathy. Expert Opin. Ther. Targets.

[30] Le Page L.M., Rider O.J., Lewis A.J., Ball V., Clarke K., Johansson E., Carr C.A., Heather L.C., Tyler D.J. (2015). Increasing Pyruvate Dehydrogenase Flux as a Treatment for Diabetic Cardiomyopathy: A Combined13C Hyperpolarized Magnetic Resonance and Echocardiography Study. Diabetes.

[31] Pooyandjoo M., Nouhi M., Shab-Bidar S., Djafarian K., Olyaeemanesh A. (2016). The effect of (L-)carnitine on weight loss in adults: A systematic review and meta-analysis of randomized controlled trials. Obes. Rev..

[32] Jeukendrup A.E., Randell R. (2011). Fat burners: Nutrition supplements that increase fat metabolism. Obes. Rev..

[33] Zhang J.-J., Wu Z.-B., Cai Y.-J., Ke B., Huang Y.-J., Qiu C.-P., Yang Y.-B., Shi L.-Y., Qin J. (2014). L-carnitine ameliorated fasting-induced fatigue, hunger, and metabolic abnormalities in patients with metabolic syndrome: A randomized controlled study. Nutr. J..

[34] Bastiaansen J.A.M., Merritt M.E., Comment A. (2016). Measuring changes in substrate utilization in the myocardium in response to fasting using hyperpolarized [1-13C]butyrate and [1-13C]pyruvate. Sci. Rep..

[35] Mikkelsen E.F.R., Mariager C.Ø., Nørlinger T., Qi H., Schulte R.F., Jakobsen S., Frøkiær J., Pedersen M., Stødkilde-Jørgensen H., Laustsen C. (2017). Hyperpolarized [1-13C]-acetate Renal Metabolic Clearance Rate Mapping. Sci. Rep..

[36] Abdurrachim D., Woo C.C., Teo X.Q., Chan W.X., Radda G.K., Lee P.T.H. (2019). A new hyperpolarized 13C ketone body probe reveals an increase in acetoacetate utilization in the diabetic rat heart. Sci. Rep..

[37] Habibuddin M., Daghriri H.A., Humaira T., Al Qahtani M.S., Hefzi A.A.H. (2008). Antidiabetic effect of alcoholic extract of Caralluma sinaica L. on streptozotocin-induced diabetic rabbits. J. Ethnopharmacol..

[38] Malatiali S., Francis I., Barac-Nieto M. (2008). Phlorizin Prevents Glomerular Hyperfiltration but not Hypertrophy in Diabetic Rats. Exp. Diabetes Res..

[39] Lee S.-I., Kim J.-S., Oh S.-H., Park K.-Y., Lee H.-G., Kim S.-D. (2008). Antihyperglycemic Effect of Fomitopsis pinicola Extracts in Streptozotocin-Induced Diabetic Rats. J. Med. Food.

[40] Chusyd D.E., Wang D., Huffman D.M., Nagy T.R. (2016). Relationships between Rodent White Adipose Fat Pads and Human White Adipose Fat Depots. Front. Nutr..

[41] Haase A., Frahm J., Matthaei D., Hanicke W., Merboldt K.-D. (1986). FLASH imaging. Rapid NMR imaging using low flip-angle pulses. J. Magn. Reson..

[42] Dodd M.S., Ball V., Bray R., Ashrafian H., Watkins H., Clarke K., Tyler D.J. (2013). In vivo mouse cardiac hyperpolarized magnetic resonance spectroscopy. J. Cardiovasc. Magn. Reson..

[43] Vanhammea L., Boogaart A.V.D., Van Huffel S. (1997). Improved Method for Accurate and Efficient Quantification of MRS Data with Use of Prior Knowledge. J. Magn. Reson..

[44] Heather L.C., Pates K.M., Atherton H.J., Cole M.A., Ball D.R., Evans R.D., Glatz J.F., Luiken J.J., Griffin J.L., Clarke K. (2013). Differential Translocation of the Fatty Acid Transporter, FAT/CD36, and the Glucose Transporter, GLUT4, Coordinates Changes in Cardiac Substrate Metabolism During Ischemia and Reperfusion. Circ. Heart Fail..

[45] Walsby-Tickle J., Gannon J., Hvinden I., Bardella C., Abboud M.I., Nazeer A., Hauton D., Pires E., Cadoux-Hudson T., Schofield C.J. (2020). Anion-exchange chromatography mass spectrometry provides extensive coverage of primary metabolic pathways revealing altered metabolism in IDH1 mutant cells. Commun. Biol..

[46] Sowell J., Fuqua M., Wood T. (2011). Quantification of total and free carnitine in human plasma by hydrophilic interaction liquid chromatography tandem mass spectrometry. J. Chromatogr. Sci..

[47] Wang X., West J.A., Murray A.J., Griffin J.L. (2015). Comprehensive Metabolic Profiling of Age-Related Mitochondrial Dysfunction in the High-Fat-Fed ob/ob Mouse Heart. J. Proteome Res..

